# Comparing Glucagon-Like Peptide-1 Receptor Agonists to Sodium-Glucose Cotransporter-2 Inhibitors in Heart Failure With Preserved Ejection Fraction: A Systematic Review

**DOI:** 10.7759/cureus.78570

**Published:** 2025-02-05

**Authors:** Moath Al-Shudifat, Bushra Sumra, Cyril Kocherry, Hina Shamim, Kiran Jhakri, Safeera Khan

**Affiliations:** 1 Internal Medicine, Faculty of Medicine, Cairo University, Cairo, EGY; 2 Clinical Research, Sanmora Bespoke Clinical Research Solutions, East Orange, USA; 3 School of Medicine, Ninewells Hospital, Dundee, GBR; 4 Pediatrics, Baqai Medical University, Karachi, PAK; 5 Internal Medicine, Shahjalal University of Science and Technology, Sylhet, BGD; 6 Internal Medicine, California Institute of Behavioral Neurosciences and Psychology, Fairfield, USA

**Keywords:** cardiovascular outcomes trials, diabete mellitus, diastolic dysfunction, diastolic heart failure, glp-1 receptor agonists, heart failure, heart failure with preserved ejection fraction (hfpef), obesity, quality of life, sglt-2 inhibitors

## Abstract

Heart failure with preserved ejection fraction (HFpEF) is a subtype of congestive heart failure distinguished by a normal ejection fraction. Comorbidities associated with its development typically include chronic conditions such as diabetes, hypertension and obesity that restrict the heart's filling pressure. Since heart failure with reduced ejection fraction (HFrEF) has been the subject of much research, physicians have always been faced with the problem of a lack of effective therapeutic interventions when treating patients with HFpEF. In recent years, there has been an increase in the number of research studies to identify effective therapeutic medication for HFpEF. Sodium-glucose cotransporter-2 (SGLT-2) inhibitors and glucagon-like peptide-1 (GLP-1) receptor agonists, which were initially developed to manage diabetes, have shown improvement in clinical outcomes in HFpEF even in the absence of diabetes. This systematic review aimed to gather and analyze evidence from randomized controlled trials and observational studies on the two drug classes. The Preferred Reporting Items for Systematic Reviews and Meta-Analyses (PRISMA) 2020 guidelines were followed in the conduct of this comprehensive systematic review. To find all relevant studies, we searched three major medical databases, including Web of Science, Cochrane Central Register of Controlled Trials (CENTRAL), and PubMed (NCBI). We have identified 13 studies on both classes of drugs, some of which have contributed to formulating current guidelines for managing HFpEF. The quality of included studies has been scrutinized using quality assessment tools, including the Cochrane Risk of Bias 2 tool and the Newcastle-Ottawa Scale tool, to ensure transparency and limit bias to lead to more reliable findings. Most studies on SGLT-2 inhibitors demonstrated a significant reduction in hospitalization rates and symptom burden, as measured by Kansas City Cardiomyopathy Questionnaire (KCCQ) scores and functional capacity, as measured by a 6-minute walk test distance. GLP-1 receptor agonists have also improved symptom scores and functional capacity, specifically in obese patients, although reductions in hospitalization rates remain unclear. Improvements in functional capacity and symptom scores were observed for both drug classes, though some metrics were not consistently statistically significant across studies. The superiority of one medication over another remains inconclusive due to a lack of trials comparing both drugs. In addition, GLP-1 receptor agonists have been more recently studied, necessitating further research on this drug class to assess long-term outcomes, efficacy in non-obese patients, and combination with SGLT-2 inhibitors.

## Introduction and background

Heart failure with preserved ejection fraction (HFpEF) is a category of heart failure characterized by a normal ejection fraction with a complex pathophysiology. The overall incidence of heart failure (HF) has decreased; however, the amplitude of decline was less in HFpEF in comparison to heart failure with reduced ejection fraction (HFrEF); this finding explains the increased HF proportion attributed to HFpEF [1]. Some studies have reported that the prevalence of HFpEF has decreased while mortality rates have not changed [1]. Other studies reported an increase in the prevalence of HFpEF, which could be a perceived increase in prevalence due to wide recognition and improved diagnosis of HFpEF in the past two decades [2]. This underscores the imperative need for well-designed epidemiological studies to identify trends in HF and the associated comorbidities. 

HFpEF development encompasses a combination of both myocardial and systemic pathologies, including impaired cardiac relaxation, dysfunction of endothelium, and systemic inflammation [1]. These pathologies are exacerbated by comorbidities such as hypertension and diabetes, which are present in 74% and 39%, respectively, of patients with HFpEF. Obesity is a prevalent comorbidity in patients with HFpEF [3,4]. Obesity increases epicardial adipose tissue, which limits the heart's diastolic function [5]. Unfortunately, obesity is also associated with multiple comorbidities that also influence the development of HFpEF. 

HFrEF has well-established diagnostic criteria and effective therapies; in contrast, HFpEF represents a challenge for clinicians due to the slow advancement in developing effective therapies. This may be attributed to the complex nature of HFpEF, as numerous comorbidities influence its development [4]. These findings have urged clinical communities to conduct more clinical trials on HFpEF to better understand the pathophysiology and determine risk factors that will help to develop better interventions.

Over the past two decades, the growing numbers of clinical trials of HFpEF have led to the emergence of new interventions. Because HFpEF represents a complex disease with different phenotypes influenced by comorbidities that patients exhibit, each has its unique pathophysiology compromising the diastolic function of the heart [4]. Consequently, the most recent interventions have focused on addressing these comorbidities that could be the root cause [4].

While there are minor differences among guidelines for confirming the diagnosis of HFpEF using algorithms and assessment scores, there’s a collective agreement that echocardiography with left ventricular ejection fraction ≥ 50% is still the cornerstone for confirming diagnosis in patients with HFpEF [6]. Brain natriuretic peptide (BNP) is usually used to support diagnosis, while other workups, such as stress echocardiography, are used in uncertain cases and cardiac magnetic resonance to identify uncommon etiologies such as amyloidosis [6]. Using these modalities in interventional studies is essential to identify phenotypes of HFpEF, which guides future research in developing targeted therapies. However, the heterogeneity of pathophysiology-related comorbidities complicates management and poses diagnostic challenges in accurately identifying underlying etiology [4,6].

Sodium-glucose cotransporter-2 (SGLT-2) inhibitors are one of the cutting-edge treatments in HFpEF that have improved clinical outcomes in both diabetic and nondiabetic individuals [7]. SGLT-2 inhibitors promote diuresis, improve glycemic control, and reduce the cardiac load, which relieves congestion and improves diastolic function [4]. Meanwhile, glucagon-like peptide-1 (GLP-1) receptor agonists have become more popular for promoting weight loss in individuals with and without diabetes. They also improve insulin sensitivity and reduce systemic inflammation [8]. Few clinical trials have investigated the utilization of SGLT-2 inhibitors and GLP-1 receptor agonists in HFpEF; clinical outcomes included various parameters, including heart failure hospitalizations, symptom burden, as measured by Kansas City Cardiomyopathy Questionnaire (KCCQ) scores, and functional capacity, as measured by a 6-minute walk test distance [7].

Compared to renin-angiotensin-aldosterone system (RAAS) inhibitors and beta-blockers (BB), SGLT-2 inhibitors and GLP-1 receptor agonists have dual metabolic and cardiovascular actions that target the multiple comorbidities that reflect the pathophysiologic heterogeneity of HFpEF [4,8]. 

This systematic review compares and explores the clinical outcomes of SGLT-2 inhibitors and GLP-1 receptor agonists in patients with HFpEF. It highlights the potential advantages of GLP-1 receptor agonists, which could be one of the limited treatment choices available for HFpEF. Although the evidence supporting their use is significant, gaps in knowledge exist. This review underscores the need for future research on GLP-1 receptor agonists to address long-term effects, efficacy in non-obese patients, and synergistic effects with SGLT-2 inhibitors. Finally, standardization of endpoints in clinical trials is needed to provide a robust comparison.

## Review

Method

Preferred Reporting Items for Systematic Reviews and Meta-analysis (PRISMA) guidelines were followed in this systematic review [9].

Search Strategy

PubMed, Cochrane Central Register of Controlled Trials (CENTRAL), and Web of Science were used due to their ability to cover a wide range of clinical trials and observational studies to ensure that high-quality, relevant evidence is included. This approach offers a thorough and adequate analysis of the literature. We used relevant articles to gather controlled vocabulary and potential synonyms to formulate a search strategy for the following concepts: heart failure with preserved ejection fraction, glucagon-like peptide-1 receptor agonists, and sodium-glucose cotransporter-2 inhibitors.

To ensure a comprehensive, reproducible, and replicable search that could be used for future updates, search queries across all databases were documented in detail for reproducibility, as shown in Tables 1-3, while two reviewers independently tested replicability. Applying no filters to the search has resulted in a larger number of studies; it has minimized the chance of excluding relevant studies and ensured the inclusion of different study designs while reducing the risk of selection bias.

**Table 1:** PubMed detailed search strategy MeSH: Medical Subject Headings

Search Number	Query	Results
1	"heart failure, diastolic"[MeSH Terms] OR "diastolic heart failure"[Title/Abstract] OR "diastolic heart failures"[Title/Abstract] OR "heart failure with normal ejection fraction"[Title/Abstract] OR "heart failure with preserved ejection fraction"[Title/Abstract] OR "heart failure normal ejection fraction"[Title/Abstract] OR "heart failure preserved ejection fraction"[Title/Abstract] OR "hfpef"[Title/Abstract]	9,740
2	"Glucagon-Like Peptide-1 Receptor"[MeSH Terms] OR "Glucagon-Like Peptide-1 Receptor Agonists"[MeSH Terms] OR "glp-1 analog"[Title/Abstract] OR "glp-1 analogs"[Title/Abstract] OR "glp-1 analogs"[Title/Abstract] OR "glp-1 receptor agonist"[Title/Abstract] OR "glp-1 receptor agonists"[Title/Abstract] OR "glp-1 receptor agonists"[Title/Abstract] OR "glp-1 analog"[Title/Abstract] OR "glp-1 analogs"[Title/Abstract] OR "glp-1 analogs"[Title/Abstract] OR "glp-1 receptor agonist"[Title/Abstract] OR "glp-1 receptor agonists"[Title/Abstract] OR "glp-1 receptor agonists"[Title/Abstract] OR "glucagon like peptide 1 receptor agonist"[Title/Abstract] OR "Glucagon-Like Peptide-1 Receptor Agonists"[Title/Abstract] OR "Glucagon-Like Peptide-1 Receptor Agonists"[Title/Abstract] OR "incretin mimetic"[Title/Abstract] OR "incretin mimetics"[Title/Abstract] OR "albiglutide"[Title/Abstract] OR "tanzeum"[Title/Abstract] OR "dulaglutide"[Title/Abstract] OR "trulicity"[Title/Abstract] OR "exenatide"[Title/Abstract] OR "byetta"[Title/Abstract] OR "bydureon"[Title/Abstract] OR "bydureon bcise"[Title/Abstract] OR "liraglutide"[Title/Abstract] OR "victoza"[Title/Abstract] OR "saxenda"[Title/Abstract] OR "lixisenatide"[Title/Abstract] OR "adlyxin"[Title/Abstract] OR "semaglutide"[Title/Abstract] OR "ozempic"[Title/Abstract] OR "wegovy"[Title/Abstract] OR "tirzepatide"[Title/Abstract] OR "mounjaro"[Title/Abstract] OR "zepbound"[Title/Abstract]	14,469
3	"Sodium-Glucose Transporter 2 Inhibitors"[MeSH Terms] OR "gliflozin"[Title/Abstract] OR "gliflozins"[Title/Abstract] OR "inhibitor sglt2"[Title/Abstract] OR "inhibitor sglt 2"[Title/Abstract] OR "sglt-2 inhibitor"[Title/Abstract] OR "sglt-2 inhibitors"[Title/Abstract] OR "sglt2 inhibitor"[Title/Abstract] OR "sglt-2 inhibitor"[Title/Abstract] OR "sglt2 inhibitors"[Title/Abstract] OR "sglt-2 inhibitors"[Title/Abstract] OR "sodium-glucose transporter 2 inhibitor"[Title/Abstract] OR "Sodium-Glucose Transporter 2 Inhibitors"[Title/Abstract] OR "sodium-glucose transporter 2 inhibitor"[Title/Abstract] OR "bexagliflozin"[Title/Abstract] OR "brenzavvy"[Title/Abstract] OR "canagliflozin"[Title/Abstract] OR "invokana"[Title/Abstract] OR "dapagliflozin"[Title/Abstract] OR "forxiga"[Title/Abstract] OR "empagliflozin"[Title/Abstract] OR "jardiance"[Title/Abstract] OR "ertugliflozin"[Title/Abstract] OR "steglatro"[Title/Abstract]	12,637
4	#1 AND #2	85
5	#1 AND #3	459
6	#1 AND #2 AND #3	21

**Table 2:** Cochrane Central Register of Controlled Trials detailed search strategy

Search number	Query	Results
1	[mh "heart failure, diastolic"] OR "diastolic heart failure":ti,ab,kw OR "diastolic heart failures":ti,ab,kw OR "heart failure with normal ejection fraction":ti,ab,kw OR "heart failure with preserved ejection fraction":ti,ab,kw OR "heart failure normal ejection fraction":ti,ab,kw OR "heart failure preserved ejection fraction":ti,ab,kw OR "hfpef":ti,ab,kw	1,931
2	[mh "Glucagon-Like Peptide-1 Receptor"] OR [mh "Glucagon-Like Peptide-1 Receptor Agonists"] OR "glp-1 analog":ti,ab,kw OR "glp-1 analogs":ti,ab,kw OR "glp-1 analogs":ti,ab,kw OR "glp-1 receptor agonist":ti,ab,kw OR "glp-1 receptor agonists":ti,ab,kw OR "glp-1 receptor agonists":ti,ab,kw OR "glp-1 analog":ti,ab,kw OR "glp-1 analogs":ti,ab,kw OR "glp-1 analogs":ti,ab,kw OR "glp-1 receptor agonist":ti,ab,kw OR "glp-1 receptor agonists":ti,ab,kw OR "glp-1 receptor agonists":ti,ab,kw OR "glucagon like peptide 1 receptor agonist":ti,ab,kw OR "Glucagon-Like Peptide-1 Receptor Agonists":ti,ab,kw OR "Glucagon-Like Peptide-1 Receptor Agonists":ti,ab,kw OR "incretin mimetic":ti,ab,kw OR "incretin mimetics":ti,ab,kw OR "albiglutide":ti,ab,kw OR "tanzeum":ti,ab,kw OR "dulaglutide":ti,ab,kw OR "trulicity":ti,ab,kw OR "exenatide":ti,ab,kw OR "byetta":ti,ab,kw OR "bydureon":ti,ab,kw OR "bydureon bcise":ti,ab,kw OR "liraglutide":ti,ab,kw OR "victoza":ti,ab,kw OR "saxenda":ti,ab,kw OR "lixisenatide":ti,ab,kw OR "adlyxin":ti,ab,kw OR "semaglutide":ti,ab,kw OR "ozempic":ti,ab,kw OR "wegovy":ti,ab,kw OR "tirzepatide":ti,ab,kw OR "mounjaro":ti,ab,kw OR "zepbound":ti,ab,kw	6,310
3	[mh "Sodium-Glucose Transporter 2 Inhibitors"] OR "gliflozin":ti,ab,kw OR "gliflozins":ti,ab,kw OR "inhibitor sglt2":ti,ab,kw OR "inhibitor sglt 2":ti,ab,kw OR "sglt-2 inhibitor":ti,ab,kw OR "sglt-2 inhibitors":ti,ab,kw OR "sglt2 inhibitor":ti,ab,kw OR "sglt-2 inhibitor":ti,ab,kw OR "sglt2 inhibitors":ti,ab,kw OR "sglt-2 inhibitors":ti,ab,kw OR "sodium-glucose transporter 2 inhibitor":ti,ab,kw OR "Sodium-Glucose Transporter 2 Inhibitors":ti,ab,kw OR "sodium-glucose transporter 2 inhibitor":ti,ab,kw OR "bexagliflozin":ti,ab,kw OR "brenzavvy":ti,ab,kw OR "canagliflozin":ti,ab,kw OR "invokana":ti,ab,kw OR "dapagliflozin":ti,ab,kw OR "forxiga":ti,ab,kw OR "empagliflozin":ti,ab,kw OR "jardiance":ti,ab,kw OR "ertugliflozin":ti,ab,kw OR "steglatro":ti,ab,kw	5,857
4	#1 AND #2	28
5	#1 AND #3	225
6	#1 AND #2 AND #3	2

**Table 3:** Web of Science detailed search strategy

Search number	Query	Results
1	TS=("diastolic heart failure” OR “diastolic heart failures” OR “heart failure with normal ejection fraction” OR “heart failure with preserved ejection fraction” OR “heart failure normal ejection fraction” OR “heart failure preserved ejection fraction” OR “hfpef”)	12,682
2	TS=("glp-1 analog” OR “glp-1 analogs” OR “glp-1 analogs” OR “glp-1 receptor agonist” OR “glp-1 receptor agonists” OR “glp-1 receptor agonists” OR “glp-1 analog” OR “glp-1 analogs” OR “glp-1 analogs” OR “glp-1 receptor agonist” OR “glp-1 receptor agonists” OR “glp-1 receptor agonists” OR “glucagon like peptide 1 receptor agonist” OR “Glucagon-Like Peptide-1 Receptor Agonists” OR “Glucagon-Like Peptide-1 Receptor Agonists” OR “incretin mimetic” OR “incretin mimetics” OR “albiglutide” OR “tanzeum” OR “dulaglutide” OR “trulicity” OR “exenatide” OR “byetta” OR “bydureon” OR “bydureon bcise” OR “liraglutide” OR “victoza” OR “saxenda” OR “lixisenatide” OR “adlyxin” OR “semaglutide” OR “ozempic” OR “wegovy” OR “tirzepatide” OR “mounjaro” OR “zepbound”)	18,326
3	TS=("gliflozin” OR “gliflozins” OR “inhibitor sglt2” OR “inhibitor sglt 2” OR “sglt-2 inhibitor” OR “sglt-2 inhibitors” OR “sglt2 inhibitor” OR “sglt-2 inhibitor” OR “sglt2 inhibitors” OR “sglt-2 inhibitors” OR “sodium-glucose transporter 2 inhibitor” OR “Sodium-Glucose Transporter 2 Inhibitors” OR “sodium-glucose transporter 2 inhibitor” OR “bexagliflozin” OR “brenzavvy” OR “canagliflozin” OR “invokana” OR “dapagliflozin” OR “forxiga” OR “empagliflozin” OR “jardiance” OR “ertugliflozin” OR “steglatro”)	16,048
4	#1 AND #2	87
5	#1 AND #3	501
6	#1 AND #2 AND #3	26

On June 30, 2024, we executed the developed searches in the three medical databases, adhering to a predetermined timeline and resource availability. A search update was scheduled if the submission for publication exceeded six months from the initial search to ensure the inclusion of the most recent studies.

Eligibility Criteria

The eligibility criteria for studies to be included in this systematic review are illustrated in Table 4. Because of the limited availability of translation resources, non-English studies were excluded. 

**Table 4:** Eligibility criteria 6MWTD: 6-minute walk test distance; GLP-1: glucagon-like peptide-1; HFpEF: heart failure with preserved ejection fraction; HFrEF: heart failure with reduced ejection fraction; KCCQ: Kansas City Cardiomyopathy Questionnaire; SGLT-2: sodium-glucose cotransporter-2

Criteria	Description
Inclusion criteria	
Population	Papers focusing on adult patients (age ≥ 18 years) and all participants diagnosed with HFpEF.
Intervention	Papers focusing on SGLT-2 inhibitors, GLP-1 receptor agonists, or both.
Outcome	Studies reporting on one or more of the following specific clinical outcomes: mortality (all-cause mortality, cardiovascular-specific mortality), hospitalization rates (heart failure-related, all-cause hospitalizations), quality of life (measured by KCCQ) and functional capacity (measured by 6MWTD).
Diabetic status	Diabetic and non-diabetic patients.
Study design	Randomized controlled trials, observational studies.
Language	Papers written and published in the English language.
Sex	Both males and females.
Exclusion criteria	
Population	Studies that included both HFpEF and HFrEF.
Study type	Animal and in vitro studies.
Data availability	Studies with limited data ability.
Study analysis	Studies with no primary data of analysis (commentaries, editorial).
Risk of bias	Studies with a high risk of bias.

Screening and Data Extraction

Records were imported to Rayyan (http://rayyan.qcri.org) to detect duplicates, which were removed after assessment based on title, authors, and date. Duplicates with slight variation in title were manually checked alongside abstracts to avoid exclusion of relevant studies. Also, using Rayaan, two authors (M.A. and B.S.) independently screened the records using title and abstract to identify potentially relevant records. Rayyan's automatic conflict flagging feature was utilized to monitor the screening process and detect conflicts between reviewers. If a conflict arose on certain records, it was confirmed first, as possible errors using Rayyan may occur. Discussion is held to reach a final decision based on the predefined population intervention outcome. Persistent disagreements were resolved by a third reviewer who was blinded to provide an unbiased assessment. 

Full texts of relevant records were retrieved, followed by data extraction from records that met all eligibility criteria. Unsuccessful retrieval attempts for full texts were documented, including efforts made to obtain access through institutional resources and alternative databases. Studies that remained unretrievable despite all efforts were marked as having no full text available to justify exclusion.

Risk of Bias Assessment

Two authors (M.A. and K.J.) conducted a quality appraisal for full-text studies that met the eligibility criteria. In the event of disagreement, an independent appraisal is carried out by a third reviewer in order to reach a consensus. Randomized clinical trials were evaluated using the Cochrane Risk of Bias 2 (RoB 2) tool [10]. Studies classified as having a high risk of bias in at least one domain based on the RoB 2 criteria will be excluded from our review. Cohort studies were evaluated using the Newcastle-Ottawa Scale (NOS) tool [11], with studies scoring below seven out of nine considered to have a high risk of bias and consequently excluded.

Results

Search Results

**Figure 1:**
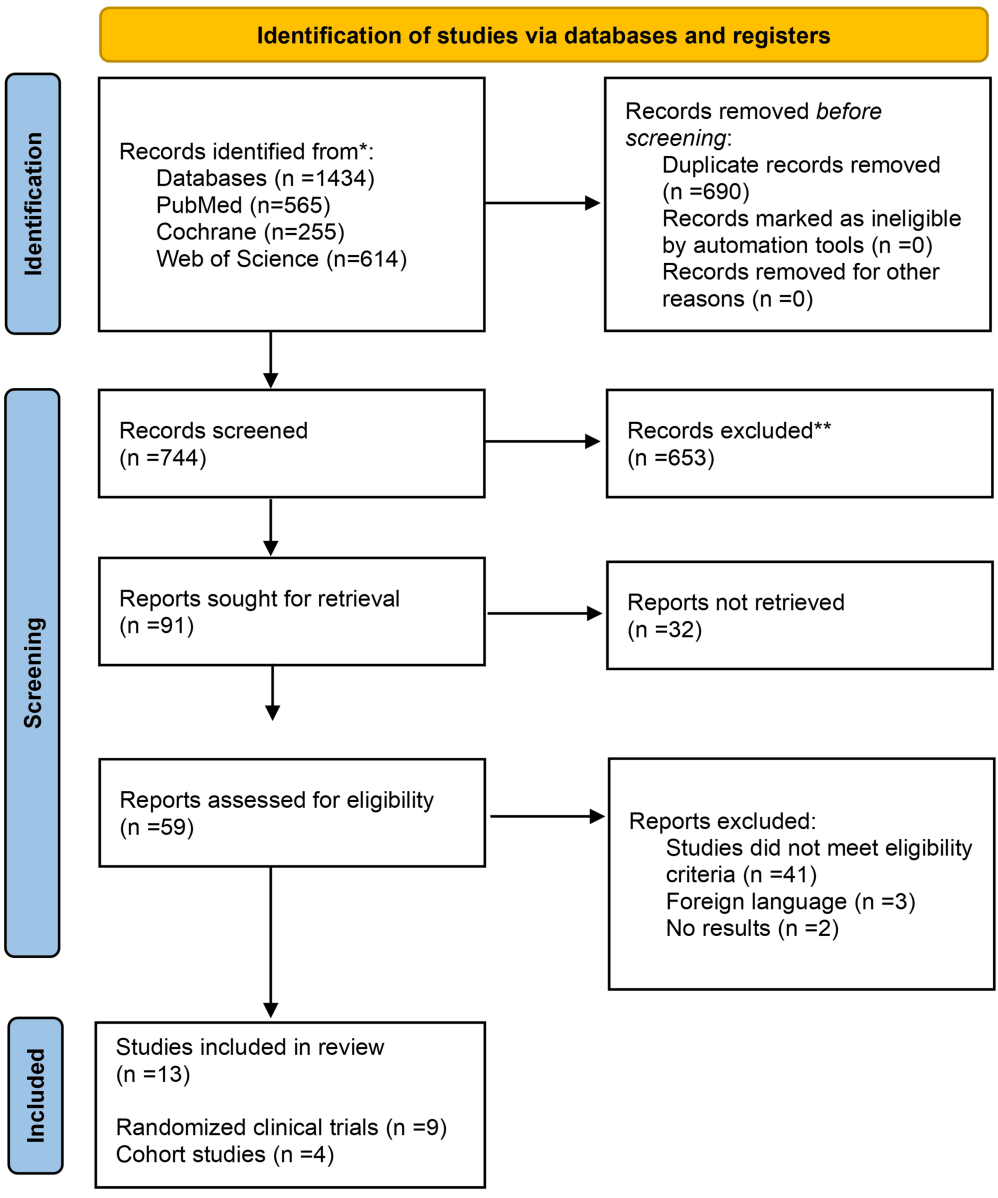
PRISMA flow diagram PRISMA: Preferred Reporting Items for Systematic Reviews and Meta-Analyses.

Initially, 1,434 results had been obtained from the database search. Throughout the process, 690 duplicates were identified and removed. The remaining 744 results were screened based on the title and abstract. After screening, 91 studies were potentially eligible for inclusion. Out of the 91, only 59 studies were retrieved in full text and assessed thoroughly for inclusion. In the end, 13 studies fulfilled the eligibility criteria and were included in this systematic review. These studies included nine randomized clinical trials and four observational cohort studies (Figure 1).

Quality Assessment

**Figure 2:**
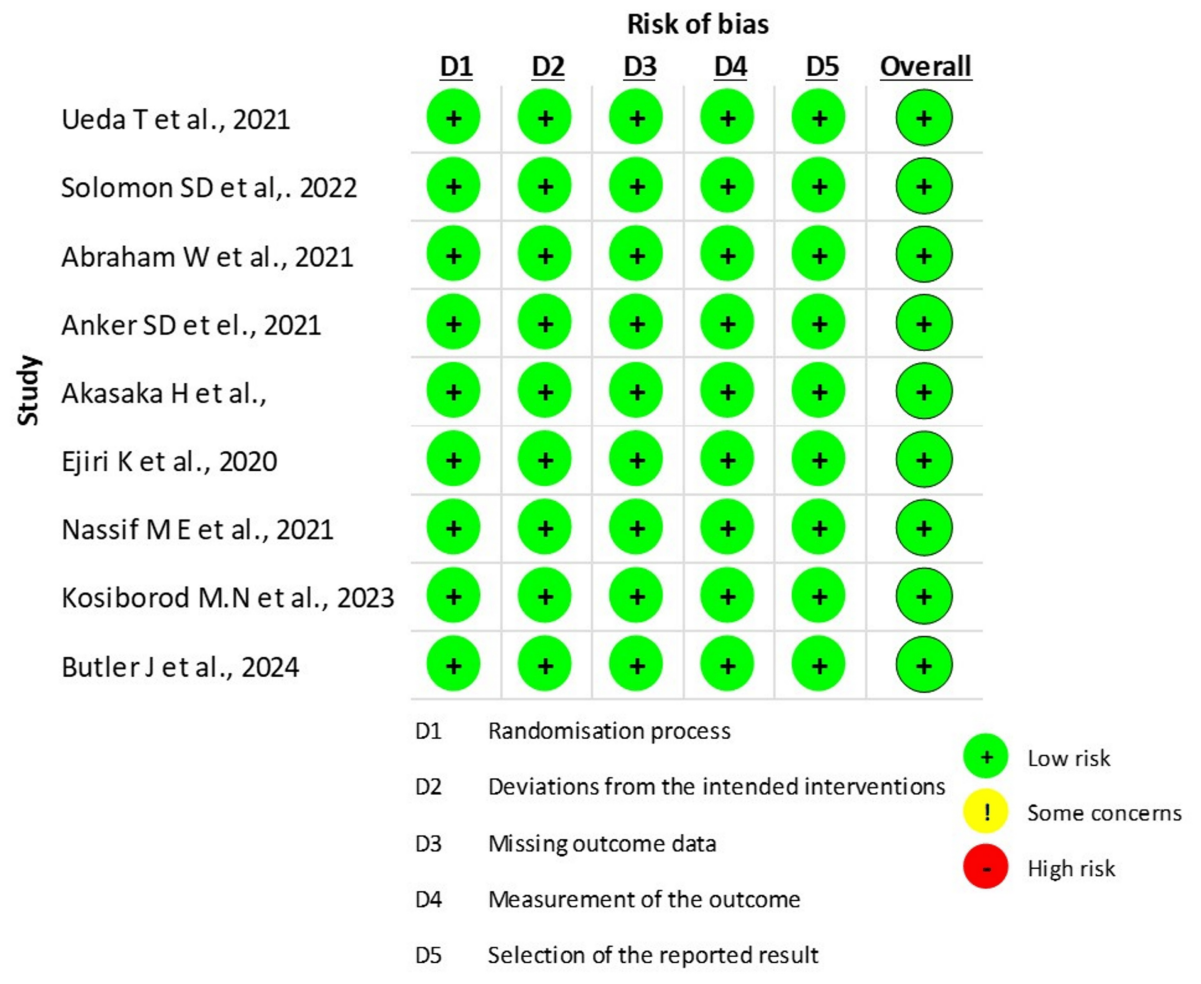
Traffic light plots used for visualization of domains in Cochrane RoB 2 tool for quality assessment of RCTs. RoB: Risk of Bias; RCT: Randomized Clinical Trial

**Figure 3:**
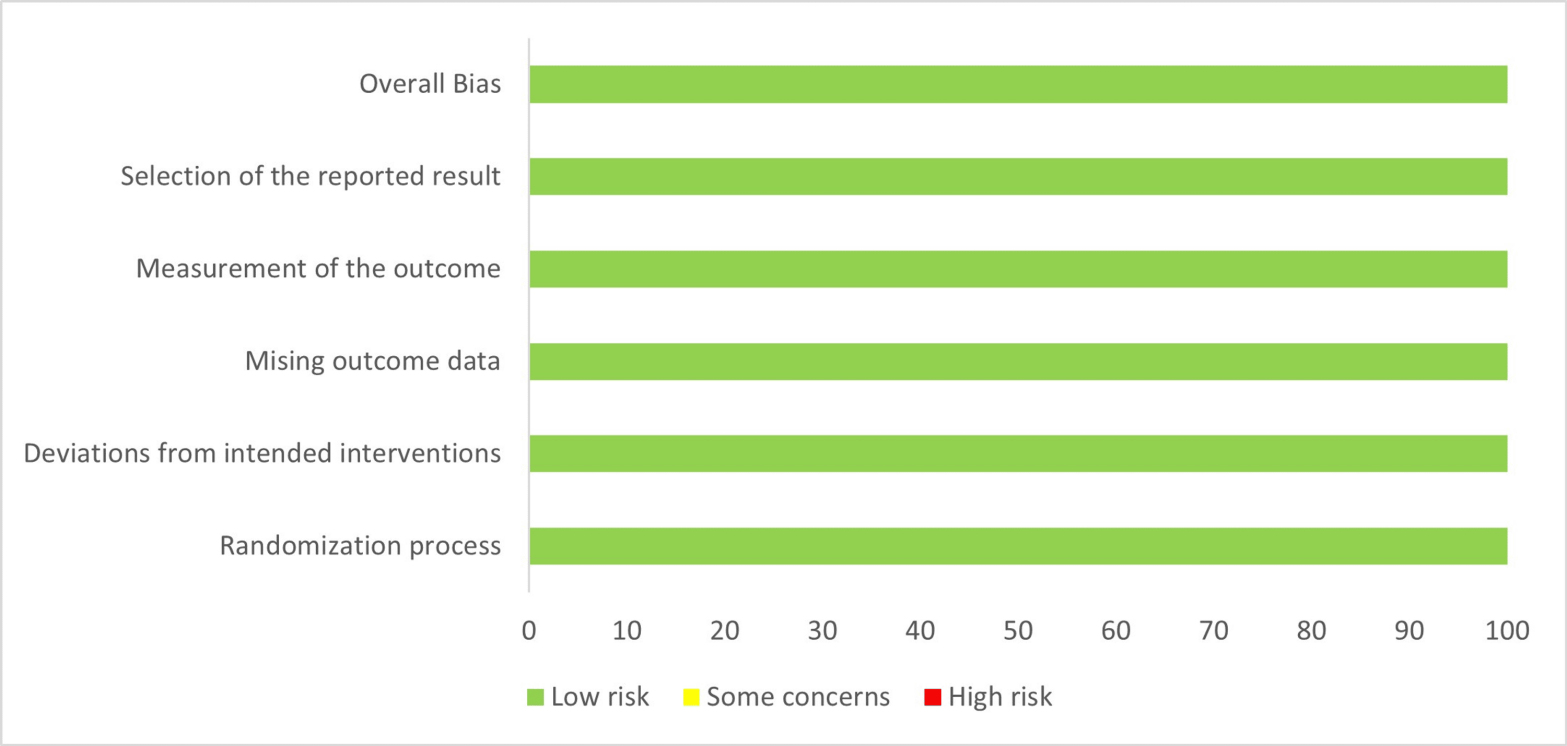
The weighted bar plots used for visualization of domains in Cochrane RoB 2 tool for quality assessment of RCTs. RoB: Risk of Bias; RCT: Randomized Clinical Trial

The RoB 2 tool assessed the risk of bias in nine randomized clinical trials using five domains illustrated in Figures 2-3 [10]. All studies have been reported to have low risk. The overall risk of bias in included randomized clinical trials was low.

The NOS tool assessed the quality of four cohort studies, as shown in Table 5 [11]. The three main domains of NOS are selection, comparability, and outcome ascertainment. Each domain had a maximum score of four, two, and three for a total score of nine. 

**Table 5:** Quality assessment of included cohort studies using Newcastle-Ottawa Scale This table presents the quality assessment of included cohort studies using the Newcastle-Ottawa Scale (NOS), which evaluates Selection, Comparability, and Outcome domains for a total score of 9. A total score of 7 or higher is considered indicative of a low risk of bias.

		Study			
		Rehman A et al., 2024 [12]	Sakai T et al., 2019 [13]	Clemmer J et al., 2023 [14]	Li W et al., 2022 [15]
Selection	Representativeness of exposed cohort	Yes	Yes	Yes	Yes
	Selection of the nonexposed cohort	Yes	No	No	Yes
	Ascertainment of exposure	Yes	Yes	Yes	Yes
	Demonstration that outcome of interest was not present at start of study	Yes	Yes	Yes	Yes
Comparability	Main factor	Yes	Yes	Yes	Yes
	Additional factor	Yes	Yes	Yes	Yes
Outcome	Assessment of outcome	Yes	Yes	Yes	Yes
	Follow up time was sufficient	Yes	No	Yes	Yes
	Adequacy of follow up	No	Yes	Yes	Yes
	Total	8/9	7/9	8/9	9/9

Some aspects have contributed to lower scores. The lack of a non-exposed group in some studies affects the ability to draw conclusions about the comparative efficacy of the intervention. Furthermore, inadequate follow-up duration has hindered the assessment of long-term outcomes such as mortality and HF-related hospitalization.

The main characteristics of the included studies are summarized in Tables 6-7. Examining these characteristics allows effective comparison of the strengths and limitations of studies to arrive at a more informed conclusion. The total number of participants in SGLT-2 inhibitor studies was 16,011, in contrast to 1,463 participants in the GLP-1RA studies. Seven studies included only participants with type 2 diabetes mellitus, while six involved both diabetic and non-diabetic participants. The age mean ranges from 60 to 75 years.

**Table 6:** Summary of the characteristics, results, and limitations of the included SGLT-2 inhibitor studies. 6MWTD: 6-minute walk test distance; AKI: acute kidney injury; BNP: B-type natriuretic peptide; BP: blood pressure; BW: body weight; CHQ-SAS: Chronic Heart Failure Questionnaire; Self-Administered Standardized format; COVID-19: coronavirus disease 2019; CVS: Cardiovascular; EF: ejection fraction; eGFR: Estimated Glomerular Filtration Rate; FMD: flow-mediated dilation; FPG: Fasting plasma glucose; HbA1c: Hemoglobin A1C; HF: heart failure; HOMA-IR: Homeostatic model assessment of insulin resistance; KCCQ-CSS: Kansas City Cardiomyopathy Questionnaire Clinical Summary Score; KCCQ-OS: Kansas City Cardiomyopathy Questionnaire Overall Summary Score; KCCQ: Kansas City Cardiomyopathy Questionnaire; LVEF: left ventricular ejection fraction; NT-proBNP: N-terminal pro-brain natriuretic peptide; NYHA: New York Heart Association; RCT: Randomized Clinical Trial; SBP: Systolic blood pressure; T2DM: type 2 diabetes mellitus

Study	Study design	Sample size	Population characteristics (age, diabetic status, LVEF, respectively)	Intervention and comparison	Follow up duration	Results/outcome		Limitations
						Primary endpoints	Secondary endpoints	
Sakai T et al., 2019 [13]	Prospective cohort	184	66.0±14.4, T2DM (100), 58±10.8	Empagliflozin, luseogliflozin, tofogliflozin	12 weeks	Significant change in echocardiographic parameters.	Significant change in FPG, HOMA-IR, and FMD.	Small sample size.
								Short follow-up period.
								All participants were Japanese.
Clemmer J et al., 2023 [14]	Retrospective cohort	2368	64 ± 14, T2DM (56), 65±10	SGLT-2 inhibitors	4 ± 2 years	SGLT-2 inhibitors improved all-cause mortality.		Single-center study.
								Some data on HF severity and cardiac parameters are missing.
Li W et al., 2022 [15]	Retrospective cohort	250	68.5, T2DM (100), more than 50%	SGLT-2 inhibitors (canagliflozin, dapagliflozin, empagliflozin) vs sitagliptin	Mean follow-up period: 295 days	Significant change in HF hospitalization.	Significant change in all-cause hospitalization and AKI rate.	Small sample size.
Ueda T et al., 2021 [16]	RCT (CANONICAL trial)	82	75.7±6.5, T2DM (100.00), 61.5±7.6	Canagliflozin vs Standard diabetic therapy	24 weeks	Significant change in BW but no significant change in BNP.	No significant change in all-cause death, CVS death, hospitalization for HF, HbA1c, echocardiography parameters, or nutritional status.	Small sample size
								Short follow-up period
								Open-label model
Solomon SD et al., 2022 [17]	RCT (DELIVER trial)	6263	71.8±9.6, T2DM (44.7), 54.0±8.6	Dapagliflozin vs Placebo	Median follow-up duration: 2.3 years	Significant change in composite risk of HF hospitalization, urgent visit for HF, and CVS death.	Significant change in total number of worsening HF events and KCCQ total symptom score, but no significant change in CVS deaths and death from any cause.	COVID-19 pandemic limited assessment of symptoms.
Abraham W et al., 2021 [18]	RCT (EMPERIAL-Preserved trial)	315	74.0, T2DM (51.1), 53.0	Empagliflozin vs Placebo	12 weeks	No significant change in 6MWTD at 12 weeks.	No significant change in 6MWTD at 6 weeks, CHQ-SAS dyspnea score at week 12, Clinical Congestion score, and NT-proBNP.	Short follow-up period.
								Enrolment of frail HF patients with baseline 6MWTD <350 m.
Anker SD et el., 2021 [19]	RCT (EMPEROR – preserved trial)	5988	71.8±9.3, T2DM (49.05), 54.3±8.8	Empagliflozin vs Placebo	Median follow-up duration: 26.2 months	Significant change in combined CVS death or HF hospitalization regardless of the presence or absence of diabetes.	Significant change in total number of HF hospitalizations and eGFR decline rate.	The discontinuation rate was high (23%) but similar in both groups.
Akasaka H et al., 2022 [20]	RCT (EXCEED trial)	68	71.9±8.0, T2DM (100), 60.9±7.0	Ipragliflozin vs Standard diabetic therapy	24 weeks	No significant change in echocardiographic parameters.	No significant change in NT-proBNP, NYHA classification, HbA1c, BP, or the frequency of adverse events.	Small sample size.
								Short follow-up period.
								All participants were Japanese, which limits the generalizability of trial results. Selection bias because participants with atrial fibrillation and valvular disease were excluded.
Ejiri K et al., 2020 [21]	RCT (MUSCAT-HF)	169	71.7±7.7, T2DM(100), 57±9.4	Luseogliflozin vs Voglibose	12 weeks	No significant change in BNP.	No significant change in echocardiographic parameters, BW, and HbA1c.	Short follow-up period.
								Open-label model.
Nassif M E et al., 2021 [22]	RCT (PRESERVED-HF)	324	70.0, T2DM(56.0), 55-65	Dapagliflozin vs Placebo	12 weeks	Significant change in KCCQ.	Significant change in 6MWTD, KCCQ-OS, KCCQ-CSS, and BW but no significant change in BNP, NTproBNP, HA1C, or SBP.	Short follow-up period.
								All participants were enrolled at sites in the United States.

**Table 7:** Summary of the characteristics, results, and limitations of the included GLP-1 receptor agonists studies. 6MWTD: 6-minute walk test distance; BW: body weight; HF: heart failure; KCCQ-CSS: Kansas City Cardiomyopathy Questionnaire Clinical Summary Score; LVEF: left ventricular ejection fraction; RCT: Randomized Clinical Trial; T2DM: type 2 diabetes mellitus

Study	Study design	Sample size	Population characteristics (age, diabetic status, LVEF, respectively)	Intervention and comparison	Follow up duration	Results/outcome		Limitations
						Primary endpoints	Secondary endpoints	
Rehman A et al., 2024 [12]	Retrospective cohort	318	69, T2DM not reported, 57	Semaglutide vs Placebo	52 weeks	Significant change in 6MWTD and BW.	Significant change in 6MWTD and CRP level, but no significant change in the hierarchical composite endpoint.	Single-center study.
								Did not evaluate clinical events such as HF hospitalizations and urgent HF visits.
Kosiborod M.N et al., 2023 [23]	RCT (STEP-HFpEF)	529	69, T2DM (0), 57	Semaglutide vs placebo	52 weeks	Significant change in KCCQ-CSS, BW percentage.	Significant change in 6MWTD, CRP level, and hierarchical composite endpoint (all-cause death, heart failure events, differences in the change in the KCCQ-CSS).	The percentage of non-white participants was low.
								Did not evaluate clinical events such as HF hospitalizations and urgent HF visits.
Kosiborod M.N et al., 2024 [24]	RCT (STEP-HFpEF DM)	616	69, T2DM (100), 56	Semaglutide vs placebo	52 weeks	Significant change in KCCQ-CSS, BW percentage.	Significant change in 6MWTD, CRP level, and hierarchical composite endpoint (all-cause death, heart failure events, differences in the change in the KCCQ-CSS).	The percentage of non-white participants was low.
								Did not evaluate clinical events such as HF hospitalizations and urgent HF visits.

The sample size is a crucial element that enhances the statistical power of the results, which also constitutes a limitation in certain studies. Trials with single-center designs may limit the generalizability of reported findings and introduce potential bias. Participants' adherence and co-intervention variability across studies may further challenge comparability. Additionally, the included studies' comparability could be affected by follow-up duration variation, which varies from 12 weeks to four years. As short follow-up duration in some studies limits the ability to evaluate long-term effects, longer duration follow-up could introduce external factors such as patient compliance and the emergence of new comorbidities. One study has mentioned that collection of data and follow-up of participants was a challenge due to the COVID-19 pandemic, and it is unclear if this limitation has affected other included studies. The lack of standardization of primary and secondary endpoints among included studies is another challenge for comparability. Therefore, similar endpoints were compared, and inconsistencies among studies were highlighted when direct comparisons were not feasible. Another limitation is the inclusion of one ethnic group in some studies and the unbalanced representation of different ethnicities in other studies, which limits the generalizability of their findings.

These limitations may result in heterogeneity among the included studies and may impact the overall strength of the conclusion drawn from this systematic review. Regardless of these challenges, these studies' findings offer critical evidence confirming the favorable clinical outcomes of SGLT-2 inhibitors and GLP-1 receptor agonists in HFpEF.

Discussion 

The categorization of heart failure by left ventricular ejection fraction has changed over the years. An ejection fraction of less than 40% defines HFrEF, while an ejection fraction of 50% or more defines HFpEF based on the latest consensus of both American and European societies [25,26]. Another category has been introduced as heart failure with mildly reduced ejection fraction (HFmrEF), with an ejection fraction of 40% to 49%. Many HFpEF studies included participants with HFmrEF, which has a distinct pathophysiology and responds better to therapies like RAAS inhibitors or BBs; this inclusion can skew study results [25,26]. For this reason, future studies should distinguish HFpEF from HFmrEF by stratifying data to ensure a clearer evaluation of treatment effects and clinical outcomes.

According to the Swedish heart failure registry data analysis, which reflects a large-scale real-world population across the heart failure spectrum based on ejection fraction, the prevalence of coronary artery disease was higher in patients with HFrEF. Comorbidities such as hypertension, atrial fibrillation, obesity, chronic kidney disease, and diabetes were more common in HFpEF patients [3]. The findings of this review similarly emphasize the significant role of these comorbidities and the importance of targeting those with a higher burden in HFpEF.

Obesity has unique mechanisms that drive the development of HFpEF that go beyond the mechanical limitation of heart diastolic function driven by increasing epicardial adipose tissue (EAT) thickness to the production of adipokines by adipose tissue with subsequent cardiac stiffness and myocardial remodeling [27,28]. In addition, obesity promotes systemic inflammation and neurohormonal activation through the overactivation of RAAS and the sympathetic nervous system, which contribute to fluid retention, vascular stiffness, and increased myocardial workload [28]. Unfortunately, the prevalence of obesity in the United States reached 42.4% in 2017-2018, which is one of the major contributing factors to the increased prevalence of HFpEF [29]. Increased incidence of HFpEF among young adults has been attributed to the phenotype of obesity-related HFpEF, which has become more widely recognized [30]. Interventions that promote weight loss have demonstrated the ability to reduce systemic inflammation, improve ventricular compliance, and alleviate symptoms, which offer a promising approach to alter the course of obesity-related HFpEF [8,27].

Diabetic cardiomyopathy represents structural and functional myocardial changes directly caused by diabetes that occur independently of other conditions like hypertension or coronary artery disease [31]. Cardiac dysfunction caused by diabetes is typically asymptomatic, and despite normal blood pressure and effective control of diabetes, 50% of patients exhibit some level of myocardial dysfunction; this is primarily diastolic dysfunction in the initial phases [32]. The mechanisms contributing to diabetic cardiomyopathy involve substrate modification, inflammatory changes, production of reactive oxygen species, and organelle dysfunction, resulting in structural changes in the myocardium, including cardiac fibrosis, hypertrophy, and compromised myocardial perfusion [31]. HFpEF with diabetes and diabetic cardiomyopathy are often confused, as both conditions share common characteristics. Diabetic cardiomyopathy represents a pathology that may progress to a multisystem clinical syndrome, presenting as either HFpEF or HFrEF [31]. However, HFpEF is much more common, especially when diabetes induces microvascular complications that impair myocardial perfusion along with endothelial dysfunction [31]. Diabetes associated with HFpEF is considered an independent predictor of hospitalization and adverse cardiovascular outcomes with high morbidity and mortality [33].

HFpEF is characterized by multiple comorbidities that do not act independently [3]. Obesity promotes systemic inflammation, and diabetes induces metabolic dysfunction, which impairs myocardial energy balance, and the combined effect accelerates cardiac stiffness and dysfunction [28,31]. These effects are compounded by chronic hypertension, which leads to left-ventricular hypertrophy and fibrosis [4]. This complex interplay between inflammatory, metabolic, and mechanical stressors highlights the need for tailored interventions to address distinct phenotypes of HFpEF [4].

Clinical Evidence on SGLT-2 Inhibitors

The Sakai et al. study is a prospective cohort study and one of the first to assess the use of SGLT-2 inhibitors in HFpEF patients through using echocardiography to evaluate the diastolic function of the left ventricle, demonstrating improvements after 12 weeks of treatment [13]. The MUSCAT-HF trial was the first randomized controlled trial (RCT) to investigate SGLT-2 inhibitors in HFpEF. Its main limitation was a small sample size, which impacted the ability to detect statistically significant changes in both primary and secondary outcomes by reducing statistical power, increasing variability, and limiting the ability to draw definitive conclusions [21]. The CANONICAL, EMPERIAL-Preserved, and EXCEED trials similarly had a small sample size [16,18,20]. Participants' and investigators' awareness of intervention in open-label trial designs has raised concerns about introducing bias, especially when subjective outcomes like symptom reporting are used where participants' perceptions could be influenced. The MUSCAT-HF and CANONICAL trials tried to minimize reporting bias with objective primary outcomes like BNP, while the EXCEED trial reduced observer bias by blinding sonographers during echocardiography [16,20,21]. On the other hand, including elderly or homogeneous populations, such as Japanese patients only in the Sakai and EXCEED studies, reduces the applicability of findings to diverse populations [13,20]. Despite the primary and secondary outcomes being nonsignificant in all of the previous trials, their findings tested hypotheses and emphasized the need for larger, well-powered, randomized clinical trials with robust blinding protocols, longer follow-up duration, and diverse participants to minimize bias and explore the underlying mechanisms of improvements in diastolic and vascular function, the potential of combination therapies with SGLT-2 inhibitors, and the long-term effects of SGLT-2 inhibitors.

The EMPEROR-preserved trial was the first large-scale RCT to investigate SGLT-2 inhibitors in patients with HFpEF; it enrolled 5,988 participants who have an ejection fraction of more than 40% and elevated N-terminal pro-brain natriuretic peptide (NT-proBNP) levels [19]. The DELIVER trial enrolled 6,263 patients with different heart failure categories, including those with HFpEF, HFmrEF, and heart failure with improved ejection fraction (HFimpEF), in which participants with reduced ejection fraction improved to more than 40% [17]. The broader inclusion offered valuable insights into treatment effects across different ejection fractions, whereas the EMPEROR-preserved trial included only participants with HFpEF and HFmrEF. Both trials demonstrated improved quality of life measured by KCCQ, reduced HF hospitalizations, and no significant reductions in cardiovascular or all-cause mortality [17,19]. Still, the DELIVER trial also showed consistent benefits across varying ejection fractions [17]. The PRESERVED-HF trial reported significant improvements in both KCCQ and 6-minute walk test distance (6MWTD), unlike the EMPERIAL-preserved trial, which had a similar sample size and follow-up duration, although it showed no significant improvement. This could be attributed to the difference in baseline characteristics of participants [18,22]. PRESERVED-HF had a higher proportion of New York Heart Association (NYHA) class III/IV patients (42% vs. 22%), greater loop diuretic use (88% vs. 72%), more females (57% vs. 43%) and African American participants (30% vs. 10%) [18,22]. These factors reflect a population with more advanced disease and greater symptom burden that is more responsive to treatment.

In 2022, American guidelines for heart failure management included SGLT-2 inhibitors as an effective treatment for managing patients with HFpEF [26]. This inclusion was based on the evidence provided by the EMPEROR-preserved trial. The recommendation was class 2a, surpassing RAAS inhibitors and mineralocorticoid receptor antagonists, which were class 2b recommendations [26]. In 2023, the European Society of Cardiology released an update on heart failure management guidelines [34]. At the time of the 2021 European guidelines, there was insufficient evidence regarding the use of SGLT-2 inhibitors in HFpEF patients; SGLT-2 inhibitors were recommended for HFrEF patients only [25]. The newest guidelines accounted for the DELIVER and EMPEROR-preserved trials as supporting evidence for recommending SGLT-2 inhibitors as a class 1A recommendation for patients with HFpEF [34].

These updates are expected to shift prescribing practices by making SGLT-2 inhibitors a first-line therapy for HFpEF to reduce HF hospitalizations, improve symptoms, and enhance the quality of life for HFpEF patients.

Finally, Clemmer et al. and Li W et al. have investigated the impact of SGLT-2 inhibitors on mortality and heart failure hospitalization, respectively, and reported improvement in both outcomes, but because they are retrospective cohort studies, they are subject to selection bias, missing data, and confounding factors. It is worth highlighting that Clemmer et al. study is distinctive as the only published study showing that SGLT-2 inhibitors enhance all-cause mortality.

Clinical Evidence on GLP-1 Receptor Agonists 

The efficacy of GLP-1 receptor agonists for weight reduction has been established, and recent years have witnessed an increase in the utilization of GLP-1 receptor agonists for promoting weight loss in nondiabetic populations [35]. The MAGNA VICTORIA trial was the first study to examine the effects of GLP-1 receptor agonists on diastolic cardiac function in diabetic patients; it is worth mentioning despite exclusion from the analysis because it did not meet the eligibility criteria as it excluded patients with cardiovascular disease, including those with HFpEF [36].

Recent large-scale studies, such as the STEP HFpEF trial, have further emphasized the role of obesity as an independent risk factor for HFpEF development [23]. A pro-inflammatory state is driven by the production of adipokines, such as tumor necrosis factor-alpha (TNF-α) and interleukin-6 (IL-6), from visceral adipose tissue, exacerbating cardiac dysfunction by promoting myocardial stiffness and impairing ventricular compliance [28,37]. These changes contribute to diastolic dysfunction by reducing the heart’s ability to relax and fill properly. Weight reduction has been shown to decrease visceral fat, which in turn reduces adipokine secretion and systemic inflammation, alleviating cardiac dysfunction [28,37]. Participants in the STEP HFpEF trial who received semaglutide showed significant reductions in weight and in C-reactive protein (CRP), demonstrating that GLP-1 receptor agonists improve both functional outcomes and quality of life through weight-dependent and anti-inflammatory mechanisms [23]. The study's conclusion was unaffected by SGLT-2 inhibitors since only 3.6% of participants were receiving this therapy, making this trial unique.

While the STEP HFpEF trial highlighted the benefits of GLP-1 receptor agonists in obesity-related HFpEF, the STEP HFpEF DM trial focused on those with comorbid diabetes and obesity, who often present with more advanced and resistant disease [24]. These patients were also more likely to be receiving SGLT-2 inhibitors, which are now the standard therapy for this population. This study has highlighted a distinct aspect of GLP-1 receptor agonists. In this trial, 32.8% of participants were on SGLT-2 inhibitors, which demonstrates that the benefits of GLP-1 receptor agonists extend beyond using SGLT-2 inhibitors. Despite the fact that weight loss was less pronounced in the STEP HFpEF DM trial, both primary and secondary endpoints remained significant, suggesting weight-independent mechanisms.

The combined use of GLP-1 receptor agonists and SGLT-2 inhibitors offers synergistic benefits by targeting both metabolic and hemodynamic dysfunctions. SGLT-2 inhibitors enhance diuresis, which reduces cardiac load and improves glycemic control, while GLP-1 receptor agonists reduce inflammation, improve insulin sensitivity, and promote weight loss [4,8]. This combination could improve functional capacity and quality of life, particularly in obese, diabetic HFpEF patients with severe metabolic and inflammatory abnormalities. Nevertheless, the potential risks of combining the two drugs include hypovolemia, electrolyte imbalances, gastrointestinal side effects, and acute kidney injury, especially in patients who are volume-depleted or have renal dysfunction [38]. Careful monitoring is recommended when combining these therapies in HFpEF patients.

While weight loss can be achieved by exercise, it remains a challenge for HFpEF patients with NYHA class IV who experience severe symptoms that limit their physical abilities. The insufficient evidence regarding exercise in this patient population excludes them from Medicare coverage for supervised exercise training and exercise-based cardiac rehabilitation [39,40]. Future research should focus on demonstrating the benefits of exercise programs for patients with HFpEF, which may support policy advocacy for future coverage revisions. GLP-1 receptor agonists and SGLT-2 inhibitors provide alternative choices for those with limited exercise tolerance.

Review Strengths 

The inaugural comparison of SGLT-2 inhibitors and GLP-1 receptor agonists in patients with HFpEF presented in this analysis fills a significant gap in comparative studies. It emphasizes the clinical outcomes of SGLT-2 inhibitors, like consistent reduction in HF hospitalization, while exploring GLP-1 receptor agonists that offer potential benefits in managing obesity-related HFpEF. In addition, it provides a direction for tailoring treatment based on patient phenotypes. Small RCTs have been included that could have been missed by other studies despite yielding nonsignificant results. These trials still played a crucial role in formulating hypotheses and treatment approaches. For example, early small RCTs like the CANONICAL and EMPERIAL-Preserved provided the groundwork for more extensive studies like the EMPEROR-Preserved and DELIVER trials, which confirmed significant clinical benefits across different patient subgroups, which formulated the current guidelines for the management of HFpEF. It also encompassed studies that underwent rigorous quality evaluation to ensure the transparency and reliability of conclusions that could be the foundation for future studies that may help to improve the approach to patients with HFpEF. Finally, no filters were applied in the search to ensure comprehensive study inclusion and minimize selection bias. Potential irrelevant or redundant studies were managed through a systematic screening process using Rayyan's conflict-flagging feature, as detailed in the Method section.

Limitations of the Review and the Included Studies 

Despite the fact that this study has shed light on innovative medical therapies for HFpEF, it should be noted that it has several limitations. These limitations may unintentionally introduce bias. First of all, the inability to access crucial databases like Scopus, Embase, and Science Direct may result in the indirect exclusion of some relevant studies. A few studies that were not in English were excluded. Some studies were limited to certain ethnicities, which limits the generalizability of their results. The primary and secondary endpoint heterogeneity of included studies was a consequence of using subjective outcomes in some studies and objective outcomes in others. Subjective outcomes, like symptom scores, can be influenced by participant perception and unblinding, which may skew the results of treatment effects. In contrast, objective markers like BNP levels provide more reliable measures of efficacy. Although all included studies had a low risk of bias, the unblinding of participants and investigators still raised concern in a few studies.

Future Research Direction 

As this review highlights the central role of SGLT-2 inhibitors in HFpEF and the promising potential of GLP-1 receptor agonists, particularly for obesity or diabetes-related HFpEF, the disparity in evidence between these drug classes underscores the urgent need for large-scale, multicenter RCTs with long-term follow-ups to evaluate GLP-1 receptor agonists and their combination with SGLT-2 inhibitors. Assessment of differences in treatment responses requires future studies to include more diverse participants, including those with varying ethnic backgrounds and different age groups, especially younger patient populations. Endpoints standardization, such as hospitalization rates, quality of life measured by KCCQ score, and biomarkers like NT-proBNP, should be emphasized to enhance comparability and reliability across studies. These measures have emerged recently as essential endpoints in most heart failure studies, and they could become the gold standard, allowing for a comprehensive assessment of both functional and clinical improvements. Robust trial designs that minimize bias and confounding factors are crucial to addressing these gaps and refining HFpEF management strategies. Since the recent SUMMIT trial was published following the database search and is not accessible in full text, it was not included in this systematic review [41]. This trial evaluated tirzepatide in obese-related HFpEF patients. It showed a significant reduction in cardiovascular death risk, worsening heart failure, and significant improvements in symptom scores. While both the STEP HFpEF and STEP HFpEF DM trials employed a hierarchical composite endpoint approach (combining all-cause death, heart failure events, and changes in KCCQ scores), the SUMMIT trial stands out for its emphasis on long-term cardiovascular event reduction and survival outcomes [23,24,41]. Future systematic reviews should consider this study, as it provides additional evidence that may guide clinical practice.

## Conclusions

An imbalance exists in the overall amount of evidence between the two drugs that favors SGLT-2 inhibitors. Important comorbidities in individuals with HFpEF and their role in pathologic progression have been covered in our systematic review. There is a growing need for tailored therapy by addressing underlying causes such as obesity, diabetes, or hypertension to slow the progression of HFpEF. SGLT-2 inhibitors offer benefits for patients with diabetes and fluid overload, while GLP-1 receptor agonists may be more effective for obese patients by promoting weight loss and reducing inflammation. Research has demonstrated the efficacy of both drug classes in enhancing functional capacity and reducing hospitalization rates, with SGLT-2 inhibitors, which are now a key component of HFpEF management. The remarkable outcomes of GLP-1 receptor agonists suggest that they could emerge as another key component but require further investigation, particularly long-term efficacy and safety in non-obese patients. Both therapy's impact on mortality rates remains unclear. Therefore, future long-term follow-up studies are necessary to address these gaps to refine future therapeutic strategies.
